# Laparoscopic pyeloureterostomy for ureteropelvic junction obstruction occurring in incomplete ureteral duplication of the solitary kidney

**DOI:** 10.1002/iju5.12268

**Published:** 2021-03-01

**Authors:** Yoshiaki Kawamura, Izumi Hanada, Taro Higure, Masayoshi Kawakami, Mayura Nakano, Nobuyuki Nakajima, Masahiro Nitta, Masanori Hasegawa, Sunao Shoji, Akira Miyajima

**Affiliations:** ^1^ Department of Urology Tokai University School of Medicine Isehara Kanagawa Japan

**Keywords:** incomplete ureteral duplication, laparoscopic pyeloureterostomy, solitary kidney, ureteropelvic junction obstruction

## Abstract

**Introduction:**

Ureteropelvic junction obstruction associated with ureteral duplication is rare, with prevalence reported to be around 2–7%. Ureteropelvic junction obstruction of the lower pole with both complete and incomplete duplex systems is a common cause of obstruction. Here, we report a case of ureteropelvic junction obstruction associated with incomplete ureteral duplication of the solitary kidney successfully treated by pyeloureterostomy.

**Case presentation:**

The patient was a 39‐year‐old woman who presented with right hydronephrosis, right back pain, and deteriorated renal function. The patient was referred to our department from the rheumatology department. Her medical history included congenital left renal hypoplasia, Sjogren's syndrome, and hyperphospholipid antibody syndrome.

**Conclusion:**

We encountered a case of hydronephrosis occurring in a solitary kidney with incomplete ureteral duplication. This case was successfully managed after pyeloureterostomy.

**Abbreviations & Acronyms**
CT: computed tomography
UPJ: ureteropelvic junction
UPJO: ureteropelvic junction obstruction

**Keynote message**

We encountered a case of laparoscopic pyeloureterostomy for UPJO occurring in incomplete ureteral duplication of the solitary kidney.

## Introduction

In addition to incomplete ureteral duplication anomaly, UPJO is a common urinary tract malformation. The probability of having a double renal pelvis and ureter has been reported to be 0.8%.^1^ However, UPJO associated with ureteral duplication is rare, and its occurrence rate has been reported to be 2–7%.^2^, ^3^, ^4^ UPJO of the lower pole, with complete and incomplete duplex systems, is a common cause of obstruction. However, true UPJO of the upper moiety is rare. This may be explained by the fact that the lower segment is anatomically the analog of a single renal system, which usually corresponds to two‐thirds of the parenchyma, at least two calyces and a true renal pelvis.^5^ Dismembered pyeloplasty is recommended for UPJO of the lower pole associated with a complete duplicated collecting system, but this procedure with incomplete ureteral duplication is difficult because the length between UPJ and confluence of both ureters is short.^6^ Here, we report a case of UPJO associated with incomplete ureteral duplication of the solitary kidney who successfully underwent pyeloureterostomy.

## Case presentation

**Fig. 1:**
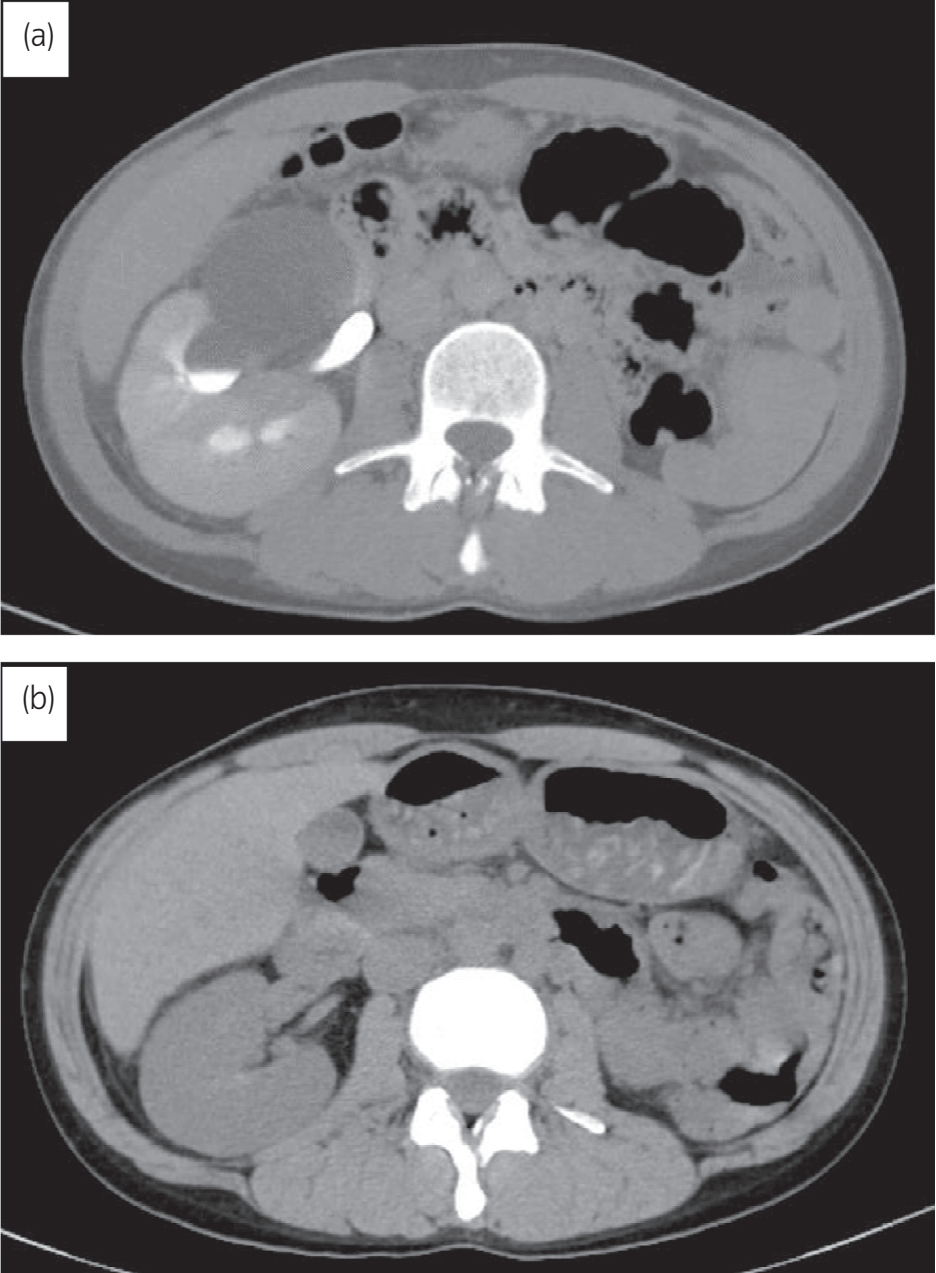
(a) CT urography showing hydronephrosis of lower pole of incomplete urinary duplication before pyeloureterostomy. (b) CT urography showing no hydronephrosis of lower pole of incomplete urinary duplication after pyeloureterostomy.

**Fig. 2:**
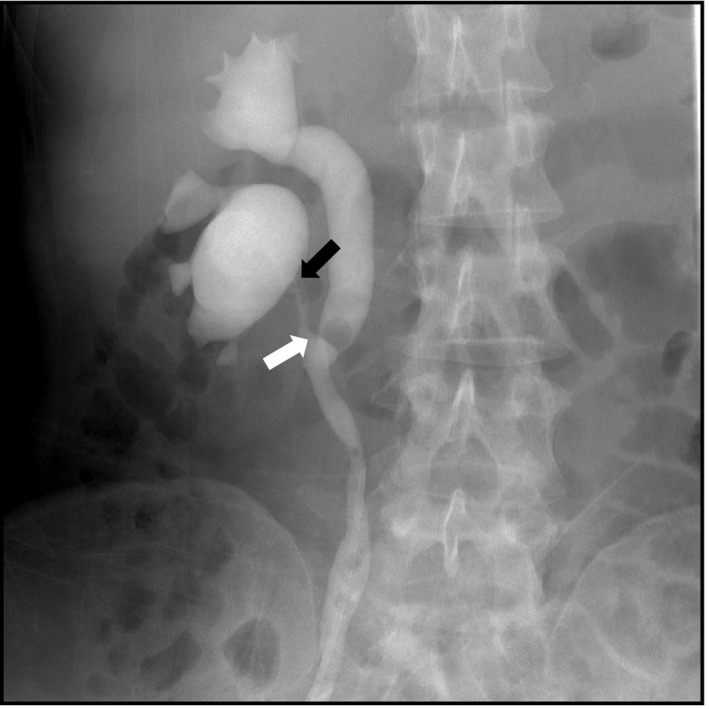
Retrograde pyelogram showing narrow ureter of the lower pole. Black arrow shows the UPJ. White arrow shows the junction of the lower and upper poles of the ureter.

The patient was a 39‐year‐old woman. She was diagnosed with right hydronephrosis with right back pain and deteriorated renal function, and she was referred to our department from Rheumatology. Her medical history included congenital left renal hypoplasia, Sjogren's syndrome, and hyper phospholipid antibody syndrome. Serum creatinine increased from 0.66 to 0.93 mg/dL. CT revealed partial right hydronephrosis, which was suspected to be an incomplete ureteral duplication (Fig. 1). Since right back pain was severe and renal function was impaired, a double‐J ureteral stent was immediately placed on lower pole before renogram. Retrograde pyeloureterography diagnosed incomplete ureteral duplication and UPJO of the lower pole in right kidney (Fig. 2). After ureteral stenting, right back pain was relieved, and renal function improved promptly.

**Fig. 3:**
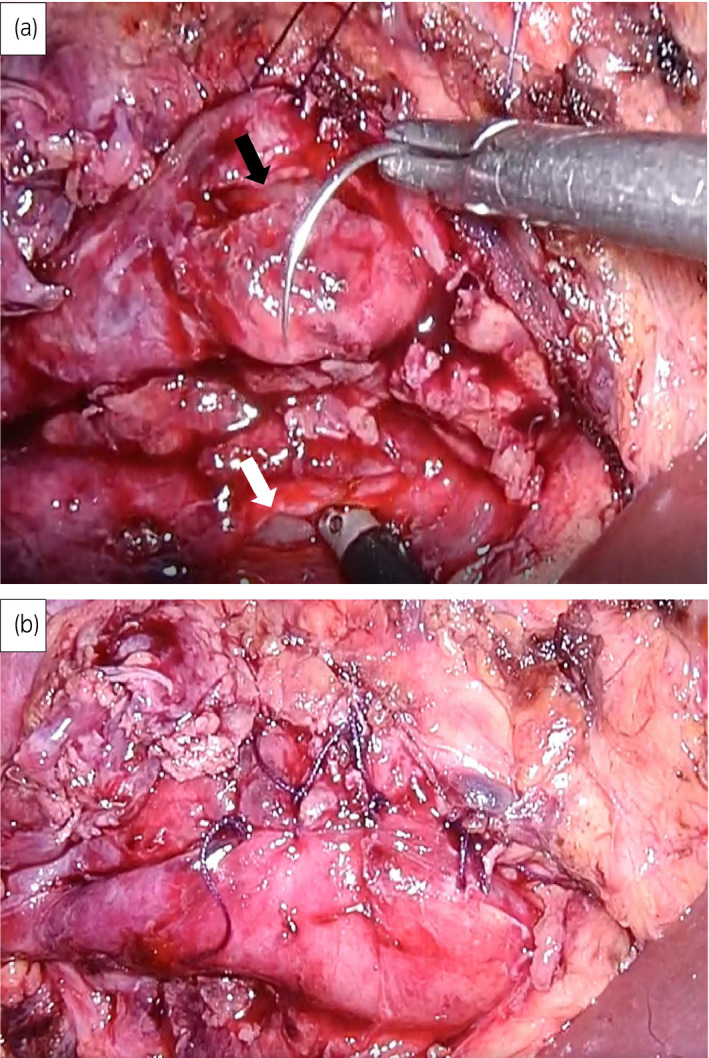
(a) Pyeloureterostomy was performed between lower pelvis (black arrow) and upper pole ureter (white arrow). (b) The picture shows anastomosis by suturing of the absorbed threads.

**Fig. 4:**
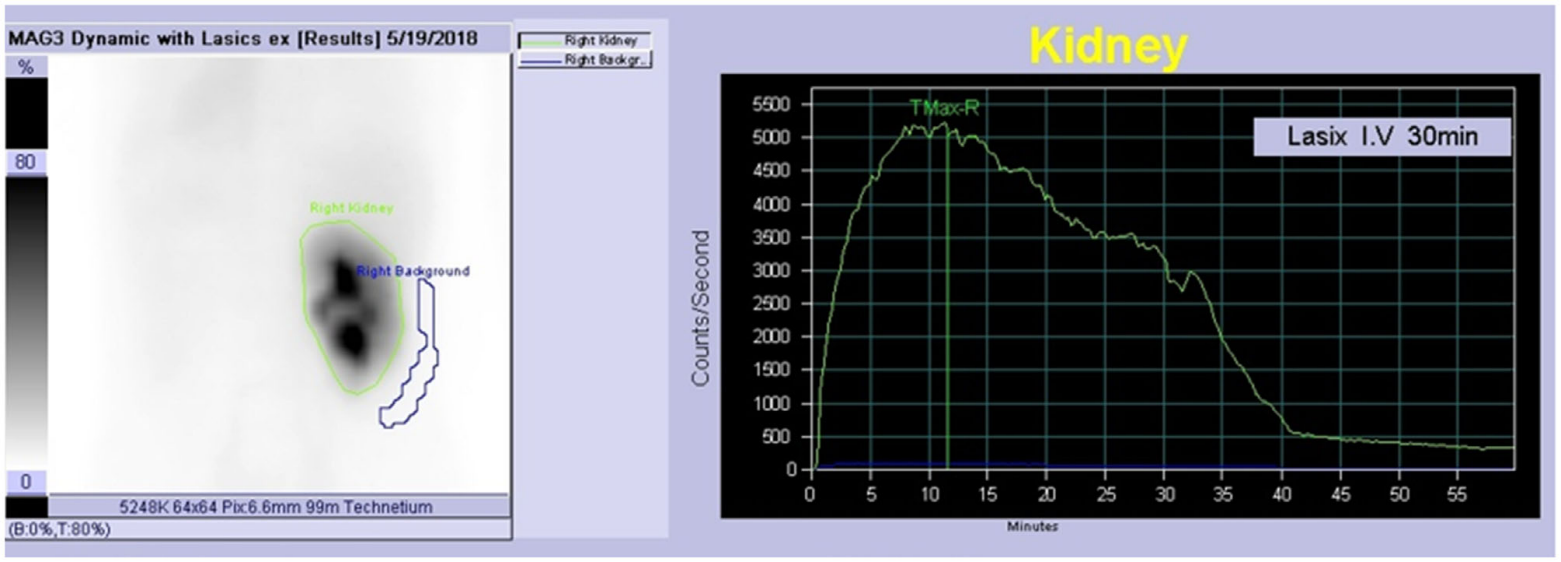
Furosemide renogram after pyeloureterostomy. Furosemide renogram before pyeloureterostomy was not performed because of the immediate double‐J stenting for elimination of patient’s back pain and improvement in renal dysfunction.

She then underwent laparoscopic right pyeloureterostomy. The adhesion around the right kidney and ureter was severe, and it was difficult to dissect around the ureter. After an intricate para‐ureteral tissue dissection, the ureter corresponding to upper renal pole and dilated renal pelvis of the lower pole were exposed. A lateral incision was made approximately 1.5 cm in the ureter to the upper pole and dilated renal pelvis of the lower pole (Fig. 3). Both ends were adapted with 3‐0 absorbed thread to form a support thread, and knot stitches were similarly applied with 3‐0 absorbed thread from the posterior wall. After the posterior wall was approximated, anterior wall was sutured with a 3‐0 absorbent thread to complete anastomosis (Fig. 3). The operation time was 5 h and 32 min, pneumoperitoneum time was 4 h and 57 min, and blood loss was 14 mL. She was discharged on the third postoperative day without any complications. The ureteral stent was removed 1 month after surgery. There was no occlusion pattern in the furosemide renogram (Fig. 4), and hydronephrosis and renal dysfunction were improved (Fig. 1).

## Discussion

To date, the surgical treatment for UPJO with ureteral duplication has been diverse and the choice for patients with UPJO tends to depend on the degree and situation of ureteral stenosis and residual renal function.^2^, ^3^, ^6^, ^7^, ^8^ Therefore, it is necessary to determine anatomical features and residual renal function before treatment. If the degree of hydronephrosis is severe (Grade 4) with thin renal parenchyma or infection, hemi‐nephrectomy is recommended.^9^ Dismembered pyeloplasty is often performed for UPJO associated with complete duplicated ureter; however, this procedure with incomplete ureteral duplication is difficult because the length between UPJ and confluence of both ureters is short.^6^ The length of the ureter from the lower pole is a substantial factor in deciding the surgical method in case of incomplete ureteral duplication, and pyeloureterostomy is suitable for incomplete duplication. Taken together, CT urography or retrograde pyelography is required for determining the surgical strategy.^10^

The present imaging study showed that distance between the lower renal pole ureter and UPJ was short, because of which pyeloureterostomy was required for management. This method is technically easier in addition to a lower risk of complications and re‐stenosis.^6^, ^7^ Moreover, this method has reflux in the lower pole, so that renal pelvic pressure flows in the corresponding ureter, leading to rapid improvement in renal function. After surgery, the present case did not show complications or renal dysfunction.

Although UPJO with ureteral duplication is often found in children, adult cases with solitary kidneys are rare. The factors inducing UPJO are reported to be crossing vessels or smooth muscle fibrosis in most cases.^11^ UPJO has several causes. Changes in the muscular layer of ureter and fibrosis are ones of those causes.^12^ In addition, adhesions around the ureter have long been known to cause hydronephrosis;^13^ however, this case may have been caused by Sjogren’s syndrome, and the surrounding tissue was difficult to dissect from UPJ. Nevertheless, robotic surgery may provide an easier way to complete the procedure in such a case.

## Conclusions

We encountered a case of hydronephrosis occurring in a solitary kidney with incomplete ureteral duplication. This case was successfully managed after performing pyeloureterostomy.

## Conflict of interest

The authors declare no conflict of interest.
